# Quantification of the glycogen cascade system: the ultrasensitive responses of liver glycogen synthase and muscle phosphorylase are due to distinctive regulatory designs

**DOI:** 10.1186/1742-4682-2-19

**Published:** 2005-05-20

**Authors:** Vivek K Mutalik, KV Venkatesh

**Affiliations:** 1 Department of Chemical Engineering and School of Biosciences and Bioengineering, Indian Institute of Technology, Bombay, Powai, Mumbai-400 076, India

**Keywords:** Glycogen, Enzyme cascade, Reciprocal regulation, Futile cycle, Glucose homeostasis, Regulatory network, Ultrasensitivity

## Abstract

**Background:**

Signaling pathways include intricate networks of reversible covalent modification cycles. Such multicyclic enzyme cascades amplify the input stimulus, cause integration of multiple signals and exhibit sensitive output responses. Regulation of glycogen synthase and phosphorylase by reversible covalent modification cycles exemplifies signal transduction by enzyme cascades. Although this system for regulating glycogen synthesis and breakdown appears similar in all tissues, subtle differences have been identified. For example, phosphatase-1, a dephosphorylating enzyme of the system, is regulated quite differently in muscle and liver. Do these small differences in regulatory architecture affect the overall performance of the glycogen cascade in a specific tissue? We address this question by analyzing the regulatory structure of the glycogen cascade system in liver and muscle cells at steady state.

**Results:**

The glycogen cascade system in liver and muscle cells was analyzed at steady state and the results were compared with literature data. We found that the cascade system exhibits highly sensitive switch-like responses to changes in cyclic AMP concentration and the outputs are surprisingly different in the two tissues. In muscle, glycogen phosphorylase is more sensitive than glycogen synthase to cyclic AMP, while the opposite is observed in liver. Furthermore, when the liver undergoes a transition from starved to fed-state, the futile cycle of simultaneous glycogen synthesis and degradation switches to reciprocal regulation. Under such a transition, different proportions of active glycogen synthase and phosphorylase can coexist due to the varying inhibition of glycogen-synthase phosphatase by active phosphorylase.

**Conclusion:**

The highly sensitive responses of glycogen synthase in liver and phosphorylase in muscle to primary stimuli can be attributed to distinctive regulatory designs in the glycogen cascade system. The different sensitivities of these two enzymes may exemplify the adaptive strategies employed by liver and muscle cells to meet specific cellular demands.

## Background

**Figure 1:**
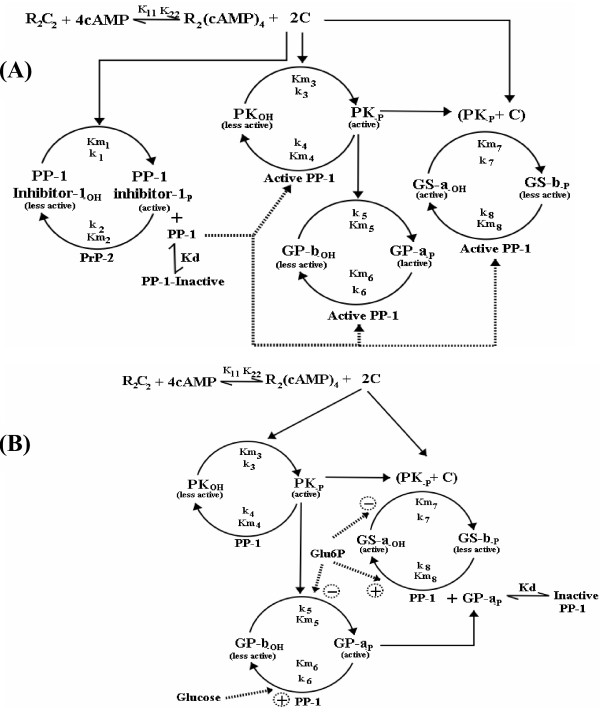
**Enzyme cascades involved in the regulation of glycogen synthesis and degradation in (A) Skeletal Muscle (B) Liver**. Nomenclature: Active enzyme form is indicated by an affix '*a' *and the corresponding inactive form is indicated by an affix '*b'*. R_2_C_2_, cyclic AMP dependent protein kinase (CAPK); C, catalytic subunit of CAPK; PP1, phosphatase-1; PrP2, phosphatases-2A; PK, Phosphorylase kinase; GP, glycogen phosphorylase; GS, Glycogen synthase; Glu6P, glucose-6-phosphate; PP1 Inhibitor-1, Inhibitor of PP1; Km_1 _to Km_8 _are Michaelis-Menten constants, k_1 _to k_8 _are rate constants, K_11_, K_22_, Kd are dissociation constants as shown in the figure. Positive and negative signs indicate the activation and inhibition of a reaction respectively. In the muscle (Fig. 1A), cAMP activated CAPK catalyzes the phosphorylation of GS, PK and inhibitor-1. Phosphorylated PK activates GP-*b*. Active phosphatase-2A is assumed to inactivate inhibitor-1, whereas PP1 catalyzes the dephosphorylation of GS, GP and PK. In liver (Fig. 1B), GP-*a *catalyzes the allosteric inactivation of GS phosphatase and inhibitor-1 does not appear to be involved in the regulation of PP1.

Signaling networks and metabolic pathways in living cells are regulated through a complex web of enzyme cascades. The regulatory architecture of these covalent modification cascades in combination with allosteric interactions determines the control of cellular processes [1,2]. A prototypical example of such an enzyme cascade system is the regulation of glycogen phosphorylase (GP) and glycogen synthase (GS), enzymes involved in glycogen degradation (glycogenolysis) and synthesis (glycogenesis) respectively [3-6]. To circumvent a futile cycle, simultaneous activation of glycogenolysis and glycogen synthesis is prevented through reciprocal regulation of glycogen phosphorylase and synthase activities by a unique regulatory network [5,6]. Although this reciprocal regulation is identical in all tissues, there are subtle differences indicating distinctive adaptation strategies in different cell types. For example, in skeletal muscle, phosphoprotein phosphatase-1 (PP1) is allosterically inactivated by inhibitor-1, whereas in the liver no such specific inhibitor has been observed [3,7]. Instead, it has been demonstrated that active GP itself plays a similar inhibitory role, regulating the GS cascade by allosterically inactivating the corresponding phosphatase [8] (Fig. 1). In liver, the phosphorylation states of GP and GS are regulated by glucose and glucose-6-phosphate, whereas in muscle, GP and GS are regulated mainly by cyclic AMP (cAMP) and calcium concentration [9]. In the absence of glycogen in the liver, i.e. under starved condition, both GP and GS appear to co-exist in an active form constituting a futile cycle, thus overcoming the reciprocal regulation existing in a normally-fed condition [10]. In the present work, we have quantified the glycogen cascade system at steady state to examine the effect of the network architecture on its performance in liver and muscle. We have also gained insights into the operation of the system in liver under fed and starved conditions. The steady state model incorporates the cascade structure, multi-step and zero-order effects and inhibitor sensitivity in response to cAMP and glucose.

The regulatory system for glycogen synthesis and breakdown mainly consists of phosphorylation and dephosphorylation of phosphorylase kinase (PK), which further regulates the activities of GP and GS [reviewed in [3-6], [9-12]] (Fig. 1). The activities of these enzymes depend on extracellular signals as hormones and on cellular-metabolic signals such as glucose and cAMP levels [5,11]. Phosphorylation of GP and GS converts them to catalytically more active (*a*-form) and inactive (*b*-form) species than their respective dephosphorylated forms. GP is activated by PK, which in-turn is activated by cAMP-dependent protein kinase (CAPK). GS is inactivated by multiple protein kinases including CAPK and PK [9]. PP1 is one of the main phosphatases catalyzing the dephosphorylation of PK, GP and GS. The regulation of PP1 activity is quite different in muscle and liver, which are the major sites of glycogenolysis and glycogenesis (Fig. 1). In liver, GS phosphatase is allosterically inactivated by active GP, whereas in muscle, PP1 is allosterically inactivated by CAPK-activated inhibitor-1 [3,5,9,12]. Thus, an increased cAMP level in the muscle cytosol not only increases the phosphorylation of PK, GP and GS, but also decreases their dephosphorylation by regulating the corresponding phosphatases. In addition to covalent modification, GP and GS are also regulated by allosteric interactions. AMP is an allosteric activator, whereas ATP and glucose-6-phosphate are allosteric inhibitors of phosphorylase-*b *[3]. Synthase-*b *is allosterically inhibited by physiological concentrations of ATP, ADP and inorganic phosphate, and is also allosterically activated by glucose-6-phosphate [9].

Experimental and theoretical quantifications [13-23] have revealed that there are significant advantages in having an interconvertible enzyme cascade structure in place of a simple allosteric interaction. These may include signal amplification, flexibility, robustness, ultrasensitivity and signal integration [22]. Ultrasensitivity has been defined as the response of a system that is more sensitive to changes in the concentration of a ligand than the normal hyperbolic response represented by the Michaelis-Menten equation [20]. The Hill coefficient has been used as a sensitivity parameter to quantify the steepness of sigmoidal dose-response curves [22]. A Hill coefficient greater than one indicates an ultrasensitive response, and a value less than one indicates a subsensitive response. The existence of ultrasensitivity in covalent modification cycles is due to the operation of enzymes in a region of saturation with respect to their substrates (termed zero order sensitivity) [14,15], involvement of the same effector in multiple steps of a pathway [15], and the presence of stoichiometric inhibitors [20]. All these requirements for ultrasensitivity appear to be fulfilled by the enzyme cascades involved in glycogen synthesis and degradation.

Edstrom and coworkers [24,25] have provided experimental proof of zero order ultrasensitivity in the muscle glycogen phosphorylase cascade. Theoretical analysis of the glucose-induced switch between phosphorylase and glycogen synthase in the liver showed the possibility of a sharp threshold in the response [26]. Furthermore, the multistep effects of cAMP in the glycogen cascade system are brought about by activation of the forward step and indirect inhibition of the reverse step (inhibition of phosphatases), thus satisfying the requirement for ultrasensitivity [27]. Although it is known that the second messenger cAMP affects five different steps in the glycogen cascades, its effective role in multistep ultrasensitivity has not been quantified. The output performance of the phosphorylase and glycogen synthase cascade in the presence of an inhibitor has also not been characterized.

The main objective of the current work was to compare the regulatory structure of the glycogen cascade system prevalent in the liver and the muscle through steady state analysis. The quantification incorporates the influences of all the effectors that regulate the output response of the glycogen cascade system. The simulation results revealed that the cascade system exhibits highly sensitive switch-like responses to changes in cAMP concentration and the output responses are surprisingly different in muscle and liver. In muscle, glycogen phosphorylase is more sensitive than glycogen synthase to cAMP, while the opposite is observed in liver. The steady state analysis indicates that, when liver undergoes a transition from starved to fed state, different proportions of active GP and GS can coexist. The transition from such a futile cycle to reciprocal regulation depends on the varying inhibition of GS phosphatase by GP and this regulation may be necessary to meet the challenges that exist under starved conditions.

## Materials and methods

The enzyme cascades involved in the regulation of glycogen synthesis and degradation in muscle and liver are schematically shown in Fig. 1A and 1B respectively. The concentrations of the metabolites ATP, AMP and PPi are assumed to be constant throughout the analysis. Allosteric regulations of GP and GS by these metabolites and effectors are also neglected. Detailed information on the set of equations and list of parameters used for the simulation are given in the *Appendix. *Most of the parameters and enzyme concentrations are taken from literature sources and the same set has been used for simulating the glycogen cascade system of skeletal muscle and liver. In the present work, the cAMP concentration is considered to be the primary input to the glycogen cascade. The fractional activations of GS (dephosphorylated form) and GP (phosphorylated form) are taken as the output responses of the glycogen system. The effects of cAMP on the enzyme cascade are mediated through activation of the allosteric enzyme CAPK. In the absence of cAMP, CAPK exists as an inactive holoenzyme, R_2_C_2_, with tightly bound subunits of the regulatory dimer R_2 _and the catalytic subunit C. However, in the presence of cAMP, R_2_C_2 _becomes activated through the binding of cAMP to the regulatory subunit and subsequent dissociation of the holoenzyme into cAMP-bound regulatory subunits and the free catalytic subunit [17]. The overall reaction scheme of CAPK activation is,

*R_2_C_2 _*+ *4*(*cAMP*) ↔ *2C *+ *R_2 _*(*cAMP*)_*4 *_    [1]

In the present work, CAPK activation by cAMP is assumed to be a stepwise dissociation of the catalytic subunits. The analytic expression for quantifying the CAPK activation is taken from Shacter *et al. *[17] and it is assumed that the complex between the catalytic subunit of CAPK and its target enzyme is negligible compared to the total concentration of CAPK. The activation of CAPK in terms of catalytic subunit formation is quantified using the following cubic equation (see *Appendix *for details):



where (R_2_C_2_)_t _denotes the total CAPK, C is the catalytic subunit, (cAMP) is the total cAMP concentration, and K_11 _and K_22 _are the dissociation constants of the first and second catalytic subunits respectively. A valid root was obtained as total CAPK catalytic subunit concentration using Eq. 2 and is taken as the input for modification of downstream target enzymes.

Figure 1A shows the schematic of the enzyme cascades involved in regulation of glycogen synthesis and breakdown in the skeletal muscle. Although dual phosphorylation of PK and multiple phosphorylation of GS have been observed *in vitro *[5,9], for simplicity we have considered a single phosphorylation site for these enzymes. To incorporate the PK and CAPK catalyzed phosphorylation of GS, it is assumed that both the enzymes form a pool before catalyzing the GS phosphorylation. Ca^+2^, which acts as another input stimulus to the system, is assumed to be present at concentrations corresponding to full activation of PK. Phosphorylated Inhibitor-1 inactivates PP1 by an allosteric reaction but it fails to inhibit phosphatase-2A. Here, we consider phosphatase-2A as a dephosphorylating enzyme of active inhibitor-1, as inhibitor-1 does not inhibit its own dephosphorylation even at saturating concentration [3].

Figure 1B shows the schematic of the glycogen cascade structure in liver. *In vitro *studies have shown that glucose-6-phosphate can stimulate dephosphorylation of GS and inhibit phosphorylation of GP-*b *and GS-*a*, whereas glucose acts as an allosteric activator of GP phosphatase [28-33]. In the present work, we have incorporated these effects along with the allosteric inhibition of PP1 by GP-*a*. It is assumed that glucose and glucose-6-phosphate influence the phosphorylation and dephosphorylation reactions by decreasing the respective Michaelis-Menten constants (see *Appendix *for equations). Glucose concentration was varied between 0.1 mM to 100 mM and the corresponding level of glucose-6-phosphate was calculated to be in the physiological range of 0.1–0.5 mM. The intracellular cAMP level is regulated by glucose concentration through hormonal signals such as glucagon. The inverse relationship between glucose and cAMP levels was incorporated to estimate the cAMP levels from the glucose concentration (details in *Appendix*)

The performance of the enzyme cascades in response to different cAMP input stimuli was analyzed by the steady state operating equation from the classic work of Goldbeter and Koshland [14]. For illustrative purposes, we present the following cubic equation, which quantifies the fractional activation inhibitor-1 (Fig. 1A) by taking all (Michaelis-Menten) complexes of a cascade into account:



where *f_1 _*= *I*/*I*_*t *_, *I*_*t *_is the total inhibitor concentration, *(PP2)*_*t *_is the total phosphatase-2A and other terms are as given in Fig. 1A. From the constraint 0 <*f_1 _*< 1, a valid root was obtained as a fractional unmodified inhibitor using Eq. 3. The fractional phosphorylated inhibitor (i.e. *I*_*p*_/*I*_*t*_) can then be obtained from the following relationship,



The operating equation for the allosteric interaction of PP1 with inhibitor-1 and phosphorylase is taken from our earlier work [34]. The following quadratic equation was used to simulate the allosteric inhibition of muscle PP1 by phosphorylated inhibitor-1, given by



where *PP1.I*_*p *_is inactive PP1 and *K*_*d *_is the dissociation constant:



where *(PP1)*_*t *_is the total PP1 and *f*_3 _is the fractional inactivated PP1 (i.e., (*PP1.I*_*p*_)/(*PP1*)_*t*_). The fractional free (active) species of PP1 (i.e., *f_4 _*= (*PP1)*/(*PP1*)_*t*_) can be estimated by *f_4 _*= *1*-*f_3_*.

In the present work, the cascade-connecting complexes are neglected. For example, complexes of PK with GP-*b *and PK with GS-*a *are neglected in the total PK balance (details in *Appendix*). The steady state operating equation for individual covalent modification cycles and allosteric interaction were sequentially connected to evaluate the output response of the cascade structure i.e. fractional modification of GP and GS to the primary input stimulus, cAMP in muscle and glucose in liver (details in *Appendix*). These equations were solved simultaneously using Matlab (The Mathworks Inc. USA) to obtain dose-response curves for fractional steady state activation of all the component enzymes at various input stimulus levels. Since most of the parameters are taken from different experimental reports, we performed the sensitivity analysis on the complete data set. To assess the sensitivity to variations in individual parameters, each parameter was varied over a 10-fold while holding all the other parameters constant.

## Results

**Figure 2:**
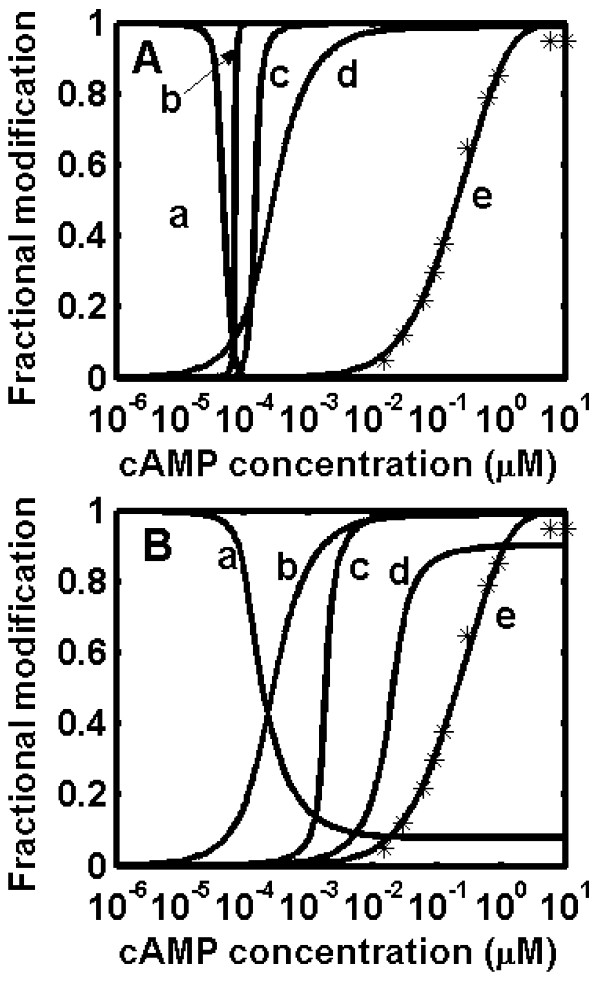
**Predicted dose-response curves in case of skeletal muscle**. The star symbol (*) represents the experimental data from Beavo *et al. *[35]. (A) Dose-response curves in the presence of inhibition of PP1 by inhibitor-1. The sensitivity of the fractional dose-response curve of glycogen synthase (curve a, Apparent Hill coefficient ~6.4), glycogen phosphorylase (curve b, ~33.8), phosphorylase kinase (curve c, ~7), inhibitor-1 (curve d, ~1.3), CAPK activation (curve e, ~1.12). (B) Dose-response curves in absence of inhibition of PP1 by inhibitor-1. The sensitivity fractional dose-response curve of Glycogen synthase (curve a, ~1.2); Glycogen phosphorylase (curve b, ~3.8); Phosphorylase kinase (curve c, ~0.8); Inhibitor-1 (curve d: ~1.3); CAPK activation (curve e, ~1.12).

The steady state model was used to obtain dose-response curves for the fractional activations of the component enzymes in glycogen synthesis and degradation. Figure 2A shows the fractional modification of GP, GS, PK, CAPK and inhibitor-1 at various concentration of cAMP in skeletal muscle. The dose-response curves show an increase in signal amplification and sensitivity as the signal propagates down the cascade. The fractional activation of CAPK at various concentrations of cAMP (curve 'e' Fig. 2A) shows a response curve with an apparent Hill coefficient () of 1.12 and the simulated results are in agreement with *in vitro *experimental studies reported by Beavo *et al. *[35]. The fractional modifications of GP and GS demonstrate ultrasensitivity with apparent Hill coefficients of 34 and 7.3 respectively (Fig. 2A). Previous experimental and theoretical studies by Edstrom and coworkers on the glycogen phosphorylase cascade reported a Hill coefficient of 2.3 in the absence of inhibitor-1 action in muscle [24]. In subsequent work, they observed that the phosphorylase cascade exhibits greater sensitivity in the presence of phosphatase inhibitor [25]. To assess the contribution of individual parameters on the output response of the system, we carried out the sensitivity analysis on the parameter set. The results indicate that the sensitivities of GP and GS display switch-like outputs in response to variation over a wide range of parameters (Table 1). Further, it can be noted that the sensitivity of the GP response is always greater than that of GS in skeletal muscle irrespective of the range considered for the parameter set. Our simulated results show that, in the absence of PP1 inhibition by inhibitor-1, the steepness of the dose-response curves and signal amplification decreased (see Fig. 2B). The fractional activations of GP and GS show apparent Hill coefficients of 3.8 and 1.9 respectively, as compared to a highly sensitive response in the presence of inhibitor action. This demonstrates that inhibitor ultrasensitivity plays a major role in imparting sensitivity to the GP and GS responses in muscle.

**Table 1:** Parametric sensitivity analysis for the glycogen cascade system. The term 'standard' indicates the parameter set used for simulation in this work and the value is indicated in parenthesis. These parameters were varied over a wide range to assess the sensitivity of the response. The star symbol indicates that the output response of a particular enzyme did not reach full activation.

**Sensitivity analysis for glycogen cascade system of skeletal muscle**						
			**Apparent Hill coefficient (Standard) to cAMP levels**			
			
**S. No.**	**Parameter (standard set)**	**Varied Range**	**GP (33)**	**GS (6.4)**	**PK (7)**	**Inhibitor -1 (1.4)**

**Rate constants (sec^-1^)**						

1	k1 (1.4)	0.14 – 14	12.2 – 48	2.4 – 17.8	13 – 3.6	1.3 – 1.3
2	k2 (0.01)	0.001 –0.1	48 – 12.2	17.8 – 2.4	3.6 – 13.9	1.3 – 1.34
3	k3 (20)	2 – 200	48 – 12.3	16.2 – 2.5	3.6 – 13.9	1.34 – 1.34
4	k4 (5)	0.5 – 50	12.3 – 48	2.5 – 16.2	13.9 – 3.6	1.34 – 1.34
5	k5 (20)	2 – 200	48.7 – 12.2	6.4 – 6.4	7 – 7	1.34 – 1.34
6	k6 (5)	0.5 – 50	12.2 – 48.7	6.4 – 6.4	7 – 7	1.34 – 1.34
7	k7 (20)	2 – 200	33.8 – 33	17.7 – 2.4	7 – 7	1.34 – 1.34
8	k8 (0.05)	0.005 – 0.5	33.8 – 33	2.4 – 17.7	7 – 7	1.34 – 1.34

**Michaelis-Menten Constants (*μ*M)**						

9	Km1 (5)	0.5 – 50	49 – 13.6	11.6 – 2.7	5.9 – 12.9	1.85 – 1.2
10	Km2 (0.7)	0.07 – 70	40.5 – 27	3.9 – 19.4	18 – 2.6	1.85 – 1.1
11	Km3 (0.4)	0.04 – 4	32 – 42.5	6 – 9	11.9 – 3.1	1.34 – 1.34
12	Km4 (1.1)	0.11 – 11	48.9 – 12	16.3 – 2.5	11.4 – 8.8	1.34 – 1.34
13	Km5 (10)	1 – 100	57.9 – 25	6.4 – 6.4	7 – 7	1.34 – 1.34
14	Km6 (5)	0.5 – 50	55 – 11.5	6.4 – 6.4	7 – 7	1.34 – 1.34
15	Km7 (15)	1.5 – 150	33.8 – 33.8	3.8 – 16	7 – 7	1.34 – 1.34
16	Km8 (0.12)	0.012 – 1.2	33.8 – 33.8	3 – 7.8	7 – 7	1.34 – 1.34

**Sensitivity analysis for glycogen cascade system of Liver**						

**S. No.**	**Parameter (standard set)**	**Varied Range**	**Apparent Hill coefficient (Standard) to glucose levels**			
	
			**GP (6.3)**	**GS (13.6)**	**PK (1.6)**	----

**Rate constants (sec^-1^)**						

1	k3 (20)	2 – 200	6 – 6	13.7 – 14	* – 2.9	----
2	k4 (5)	0.5 – 50	6 – 6	14 – 13.7	* – 2.9	----
3	k5 (20)	2 – 200	5.3 – 5.4	21 – 14.1	1.6 – 1.6	----
4	k6 (5)	0.5 – 50	5.4 – 5.4	14.1 – 21	1.6 – 1.6	----
5	k7 (20)	2 – 200	6.3 – 6.3	13 – 20.1	1.6 – 1.6	----
6	k8 (4)	0.4 – 40	6.3 – 6.3	20.1 – 13	1.6 – 1.6	----

**Michaelis-Menten Constants (*μ*M)**						

7	Km3 (0.4)	0.04 – 4	6.3 – 6.2	13.6 – 13.4	4 – *	----
8	Km4 (1.1)	0.11 – 11	10.6 – 5.4	14.5 – 13.8	* – 2.9	----
9	Km5 (10)	1 – 100	11.2 – 5	12.2 – 18.7	1.6 – 1.6	----
10	Km6 (5)	0.5 – 50	8 – 3.8	27 – 11.3	1.6 – 1.6	----
11	Km7 (15)	1.5 – 150	6.3 – 6.3	20.5 – 13	1.6 – 1.6	----
12	Km8 (0.12)	0.012 – 1.2	6.3 – 6.3	13.9 – 12.3	1.6 – 1.6	

**Figure 3:**
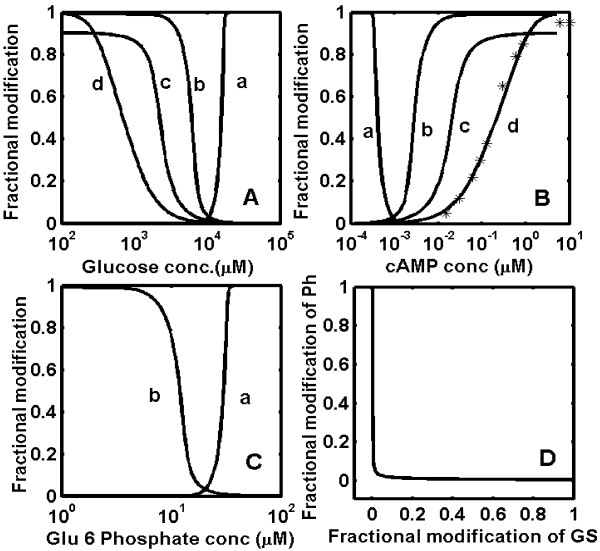
**Simulated results of glycogen cascade system in liver, incorporating glycogen synthase phosphatase inhibition by phosphorylase-*a***. (A) Fractional modification of enzymes at various concentration of glucose. The sensitivity of the fractional dose-response curve of glycogen synthase (curve a, ~13.6), phosphorylase (curve b, ~6.3), phosphorylase kinase (curve c, ~1.6), CAPK (curve d, ~1.12). (B) Fractional modification of enzymes at various concentrations of cAMP. The sensitivity of fractional dose-response curve of glycogen synthase (curve a, ~6.8), phosphorylase (curve b, ~3.2), phosphorylase kinase (curve c, ~1.6), CAPK (curve d, ~1.12). (C) Fractional modification of enzymes at various concentrations of glucose-6-phosphate. The sensitivity of the fractional dose-response curve of glycogen synthase (curve a, ~14.2) and phosphorylase (curve b, ~6.4). (D) Fractional modification of phosphorylase as a function of glycogen synthase demonstrating reciprocal regulation. The dissociation constant (Kd) of phosphorylase-a binding to glycogen synthase phosphatase is taken as 0.002 *μ*M.

The analysis was extended to the glycogen cascade system in liver. The coordinated changes in the phosphorylation of PK, GP and GS are under the influence of cAMP, glucose and glucose-6-phosphate concentrations (Fig. 1B). Figure 3 shows the predicted performance of the glycogen cascade system in liver at different concentration of glucose, glucose-6-phosphate and cAMP. The results are surprisingly different from those obtained in muscle. Figure 3A shows that the fractional activation of GS exhibits a steeper response with an apparent Hill coefficient of 13.6, while GP demonstrates a response with an apparent Hill coefficient of 6.3 with respect to glucose. The response sensitivity of GS was found to be highly dependent on the GP-*a *concentration. This result is seems to be in agreement with a recent study showing that hepatic glycogen synthesis and glycogen synthase activity is highly sensitive to phosphorylase activity [36]. Because of the stronger binding between GP-*a *and GS phosphatase, GS becomes activated only when the GP-*a *levels drop below 1%. This inverse switching between the inactivation of GP and activation of GS occurs at a glucose concentration of ~10 mM. This result is in agreement with the experimental observation that GS becomes activated once GP-*a *inhibition of GS phosphatase becomes negligible, and this shift in activity occurs after meals when the glucose concentration rises above 10 mM [10,37]. Sensitivity analysis of the parameter set indicated that the fractional modifications of GS and GP to glucose levels display switch-like outputs (Table 1). It was noted that the sensitivity of the GS response is always greater than that of GP in liver irrespective of the range considered for the parameter set. The simulated dose-response curves for fractional activation of GP-*a *and GS-*a *at various concentrations of cAMP also show an ultrasensitive response. The threshold concentration of cAMP required to activate GP and inactivate GS is higher in liver (~1 nM) than in muscle (~0.01 nM). The dose-response curve for fractional modification of the enzymes with respect to glucose-6-phosphate demonstrates that the switching between GP and GS occurs at 20 *μ*M with an ultrasensitive response (Fig. 3C). Our result is consistent with earlier observations showing an inverse correlation between the activity of GP-*a *and the concentration of glucose-6-phosphate [33]. Similarly, a direct correlation exists between GS-*a *levels and glucose-6-phosphate concentration. The threshold activation of phosphorylase and glycogen synthase is shown explicitly in Fig. 3D by plotting the active fraction of synthase against the active fraction of phosphorylase. GS is activated only when GP is mostly inactive, demonstrating the inverse relationship between the activities of the two enzymes.

**Figure 4:**
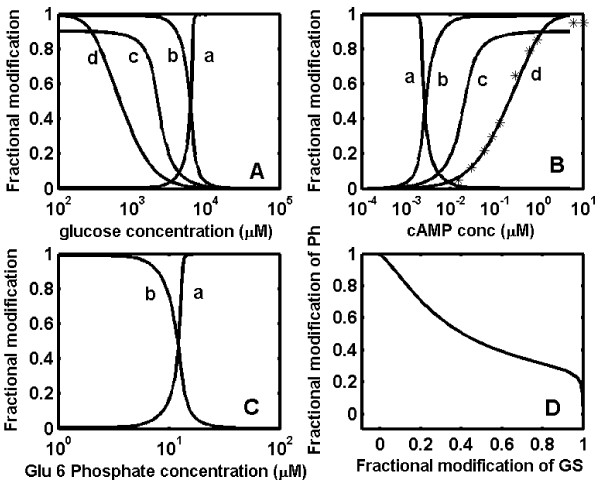
**Simulated results of glycogen cascade system in liver under starved conditions**. (A) Fractional modification of enzymes at various concentrations of glucose. The sensitivity of the fractional dose-response curve of glycogen synthase (curve a, ~10.4), phosphorylase (curve b, ~6.2), phosphorylase kinase (curve c, ~1.6), CAPK (curve d, ~1.12) (B) Fractional modification of enzymes at various concentrations of cAMP. The sensitivity of the fractional dose-response curve of glycogen synthase (curve a, ~5.2), phosphorylase (curve b, ~3.1), phosphorylase kinase (curve c, ~1.6), CAPK (curve d, ~1.12) (C) Fractional modification of enzymes at various concentrations of glucose-6-phosphate. The sensitivity of the fractional dose-response curve of glycogen synthase (curve a, ~10.5) and phosphorylase (curve b, ~6.4). (D) Fractional modification of phosphorylase as function of glycogen synthase. The dissociation constant (Kd) of phosphorylase-*a *binding to glycogen synthase phosphatase is taken as 2 *μ*M (~1000 fold higher Kd than used to simulate results shown in Fig 3). Appreciable amounts of both glycogen synthase and phosphorylase exist in such a fasted state.

The inhibition of GS phosphatase by GP-*a *depends on glycogen concentration in liver and it has been shown that a minimal concentration of glycogen is essential for this inhibition [38,39]. To simulate the fasted or glycogen depleted state in liver, the steady state analysis was repeated with the inhibition constant of GP-*a *reduced. The simulated results (Fig 4) show that, at a 1000 fold decrease (Kd value of 2 *μ*M) in the inhibition of GS phosphatase by GP-*a*, the liver may have appreciable amounts (about 50%) of both GP-*a *and GS-*a *at 4 to 9 mM glucose. This result is in agreement with the experimental observation reported by Massillon *et al. *[38]. We observe that this decrease in the steepness of the GS response curve is due to reduction in the phosphatase inhibition by GP-*a*. A decrease of similar extent in the ultrasensitivity of the GS response was observed with respect to cAMP and glucose-6-phosphate (see Fig. 4B and 4C). Furthermore, plotting the active fraction of GP as a function of the active fraction of GS demonstrates the absence of reciprocal regulation in the fed state (Fig. 4D).

**Figure 5:**
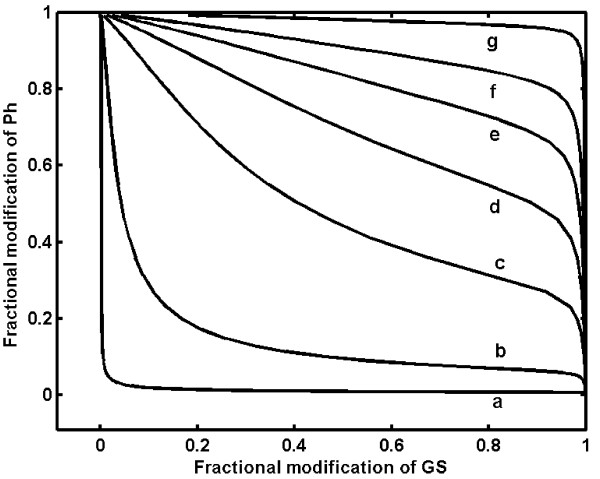
**Variable fractional levels of active phosphorylase-a and synthase-a in the liver under fasted (glycogen depletion) state**. The dissociation constant of phosphorylase-a binding to glycogen synthase phosphatase was varied from 0.002 *μ*M to no-inhibition (very high Kd), to simulate the metabolic transition from fasted to fed state. The values of dissociation constants (Kd) used are, curve a: 0.002 *μ*M; curve b, 0.2 *μ*M; curve c, 2 *μ*M; curve d, 5 *μ*M; curve e, 10 *μ*M; curve f, 20 *μ*M; curve g, very high dissociation constant (~10^6^). The active fraction of glycogen synthase and phosphorylase coexist in liver in the no-inhibition state (starved condition), while simultaneous activation of phosphorylase and inactivation of synthase is seen in liver in the fed state. The fractional active form of glycogen synthase and phosphorylase varies over a wide range between these operations.

The exact percentage reduction in the inhibition of GS phosphatase by GP-*a *is unknown. When liver undergoes a metabolic shift from completely starved to fed state, the inhibition of GS phosphatase can vary over a wide range. This was simulated by changing the inhibition constant (Kd) of GS phosphatase from 0.002 *μ*M to a very high Kd value to represent no inhibition. These results are shown in Fig. 5 as a plot of the active fraction of GP against the active fraction of GS at different inhibitor constants. In the complete absence of inhibition, both GS and GP exist in 100% active states indicating a futile cycle (curve 'g' Fig. 5). In such a state, the cells would not accumulate glycogen due to continuous glycogenolysis by GP-*a*. In the fed state, i.e. in the presence of appreciable amounts of glycogen in the liver, the inhibition of GS phosphatase by GP-*a *is high and a reciprocal regulation of GP and GS activity is observed (curve a, Fig. 5). Different proportions of active fractions of GP-*a *and GS-*a *can coexist when conditions change from starved to fed state, owing to varying net glycogen concentrations in the liver (curves b-f, Fig. 5).

## Discussion

The coordinated regulation of glycogenolysis and glycogenesis in the liver and the skeletal muscle is dependent on a network of interacting enzymes and effectors that determine the fractional activation of GP and GS [3-6,9-12]. In the present work, the cascades involved in the regulation of glycogen synthesis and breakdown were analyzed at steady state to gain an insight into the inherent design principle of the regulatory cascades existing in muscle and liver. Using experimental data from the literature for rate and Michaelis-Menten constants, the simulation results revealed that, in muscle, the response of GP to cAMP input is more highly sensitive (~34) than that of GS (~6.5), whereas in the liver, the GS sensitivities to glucose (~13.6) and cAMP (~6.8) are high compared to that of GP (~6.3 for glucose and ~3.2 for cAMP). The sensitivity analysis indicated that this differential performance of GS and GP in liver and muscle is due to the presence of a distinctive regulatory design and not to selection of a particular parameter set. CAPK-activated inhibitor-1 inhibits PP1, which is a major dephosphorylating enzyme in muscle, whereas GP-*a *inhibits GS phosphatase in liver, representing this distinctive design. The simulation results indicate that the response sensitivity of GS with respect to glucose and cAMP is highly dependent on the GP concentration in liver. Similarly, the sensitivities of the PK, GP and GS responses are dependent on inhibitor-1 concentration in muscle. The ultrasensitive response of these enzymes may be attributed to the known system-level mechanisms, namely, multistep ultrasensitivity due to cAMP, inhibitor ultrasensitivity due to phosphatase inhibitor and zero order effects due to the pyramidal relationship in enzyme component concentrations. However, the significance of this switch-like response of GP in muscle and GS in liver is unclear. It can be argued that glycogen breakdown in muscle has to be sensitive to the second messenger cAMP in order to meet the urgent requirement for glucose during exercise or the fight and flee response. Similarly, glycogen synthesis in liver has to be sensitive to blood glucose concentration, so that GS can start synthesizing glycogen whenever the blood glucose concentration increases beyond a toxic level.

In muscle, the ultrasensitive response of GP can be directly attributed to the presence of zero order effects (GP concentration about ~70 *μ*M) and compounded by the inhibitor ultrasensitivity imparted by inhibitor-1. Such a direct effect is not observed in GS owing to its minimal zero order effects (GS concentration about ~3 *μ*M). The primary stimulus, cAMP, not only increases the phosphorylation of PK, GP and GS, but also indirectly decreases their dephosphorylation through inhibitor-1. In liver, the ultrasensitive response of GS can be attributed mainly to the inhibitor ultrasensitivity caused by GP on the GS modification cycle. In this case, the zero order effect actually resides in the GP cascade, which transmits it to the GS cycle by inhibiting the dephosphorylation reaction. Furthermore, the stimulatory effect of glucose on dephosphorylation of GP-*a*, the inhibitory effect of glucose-6-phosphate on phosphorylation of GP-*b *and GS-*a*, and the stimulation of GS dephosphorylation by glucose-6-phosphate, enhance the sensitivity of GS. Thus, the ultrasensitivity of GS in liver is brought about by the combined action of the multistep effects of cAMP, the inhibition of GS phosphatase by active GP and the influence of glucose and glucose-6-phosphate concentration.

It is noteworthy that the simultaneous activation and deactivation of GP and GS respectively in muscle and liver results in reciprocal regulation of these enzymes by the primary stimulus. This reciprocal regulation, although identical in all tissues, still imparts a distinctive adaptive strategy in different cell types owing to subtle differences in the network. For example, the inhibition of GS phosphatase by GP in liver can compromise the reciprocal regulation in the absence of liver glycogen (i.e. starved state), while in muscle the reciprocal regulation cannot be compromised owing to an independent inhibitor-1. Our simulation of the glycogen cascade system under starved condition demonstrates that the sensitivity of GS reduces because of the reduction in inhibitor ultrasensitivity caused by GP. The percentage reduction in the inhibition of GS phosphatase is unknown. It is possible that when the liver undergoes a transition from starved to fed state, GS phosphatase can experience varying degrees of inhibition by GP. This results in a shift from a highly futile cycle with no inhibition to reciprocal regulation in the fed state. This causes GS to be always active, while GP is active only in the starved state in the presence of high glucose (see Fig. 5).

Hallenbeck and Walsh [40] observed that, if the quantity of phosphorylase sequestered in the glycogen particle compartment of rabbit muscle is taken into account, then the local concentration of GP can be very high (up to 2–5 mM). Furthermore, GP interaction with the glycogen particle is known to lower the Michaelis-Menten constants of PK and PP1, thus enhancing the zero order effects further [29,25]. Considering these observations, Meinke and Edstrom [25] estimated an apparent Hill coefficient of 51 for the activation of 3.5 mM phosphorylase. Our simulation results show that at 3.5 mM phosphorylase the system can actually show a highly ultrasensitive response with an apparent Hill coefficient as high as 200 (results not shown). This apparent Hill coefficient value is far higher than any known ultrasensitive system or any of the cooperative enzymes. Though the utility of such a highly sensitive response *in vivo *is unclear at present, various observations indicate that the multi-enzyme cascade system has the potential to exhibit higher sensitivity.

Signaling by hormones such as glucagon and epinephrine is known to elicit responses within a fraction of a second, incorporating amplification of the input signal and enhanced sensitivity to allosteric effectors [2,3,27,41]. It has also been shown, in the contraction of resting muscle, that GP-*b *is converted to GP-*a *within a second followed by immediate initiation of glycogenolysis [3]. Such rapid and sensitive responses are known to be the characteristic behavior of enzyme cascades with progressive increase in enzyme concentration down the cascade [2]. This effect can also be brought about by the opposing action of the same effector on modifying and demodifying enzymes [18] and the presence of a stoichiometric inhibitor [20]. It appears that living systems use these ultrasensitive regulatory mechanisms to coordinate multiple input signals, show varied responses to different signals, exhibit rapid responses at an invariably low stimulus concentration [2,3,27] and, most importantly, use negligible amount of cellular energy [42,43].

Theoretical quantification of a regulatory system as presented here reveals insights into system level properties. Ultrasensitivity, signal amplification, flexibility in operation and signal integration are all system level properties, and are not apparent in isolated components. These properties can be studied by connecting different functional units and defining the quantitative relationship between different components of a system. Our simulation results revealed that the switch-like responses of GP and GS in liver and muscle are comparable with that of the MAPK cascade in *Xenopus *oocytes [21]. At the metabolic level, GP and GS are also regulated by calcium levels and feedback loops constituted by effectors such as ATP, AMP, cAMP, glucose and glycogen [3-6,9-12]. Furthermore, GS and PK are known to have multiple phosphorylation sites [5,9]. Regulatory networks made up of multiple feedback loops and multiple phosphorylation cycles, as seen in the activation of maturation-promoting factor and the MAP kinase cascade during oocyte maturation [44,45], can yield multiple steady state responses. Although we have not incorporated the overall regulatory network, our analysis suggests that the enhanced sensitivity observed in the glycogen cascade system may act as a selective pressure in evolution favoring tissue-specific adaptive strategies and compartmental regulatory modules.

## The abbreviations used are

GP: Glycogen Phosphorylase;

GS: Glycogen Synthase;

cAMP: cyclic AMP;

PP1: Phosphoprotein Phosphatase-1;

PK: Phosphorylase Kinase;

CAPK: cAMP dependent Protein Kinase;

## Competing interests

The author(s) declare that they have no competing interests.

## Authors' contributions

VKM and KVV conceived and designed the experiments. VKM performed the experiments. VKM and KVV analyzed the data. VKM and KVV conceptualized the manuscript. All authors have read and approved the final manuscript.

## Appendix

The following equations were solved in Matlab (The Mathworks Inc. USA) to obtain dose-response curves for fractional steady state activation of component enzymes at various cAMP levels. The steepness of these stimulus dose-response curves can be approximated using the Hill equation. The global output response (fractional modification of phosphorylase and glycogen synthase) can then be quantified in terms of apparent Hill coefficients and half saturation constants, with respect to the input stimulus concentration. Here, the half saturation constant is the amount of input stimulus required for 50% fractional modification of the corresponding protein kinase. Thus, the half saturation constant indicates a mid-point on the unmodified to modified kinase transition curve. The apparent Hill coefficient can also be calculated by estimating the primary input concentration required for 10% to 90% modification of the target enzyme by using the following equation:



where *I*_0.1 _and *I*_0.9 _are the input concentrations required for 10% to 90% modification of target enzyme and  is the apparent Hill coefficient. In the following section we detail the solution strategy employed in simulations.

The following equations are derived for the glycogen cascade system schematically shown in Fig 1.

### (I) Activation of cAMP dependent protein kinase (CAPK) by cAMP

Two cAMP molecules bind to each R subunit of CAPK (R_2_C_2_) through an infinitely cooperative mechanism and this results in stepwise dissociation of the catalytic subunit [17]





where *K_11 _*and *K_22 _*are the dissociation constants and *C *is a catalytic subunit.





Mass balance on catalytic subunit yields

[*C*_*t*_] = *2*[*R*_*2*_*C*_*2*_] + [*R*_*2*_*C*(*cAMP*)_*2*_] + [*C*]     [A6]



Using equations [A6] and [A7], we obtain a cubic equation for the active catalytic subunit *C*.



where [R_2_C_2_]_t _denotes total CAPK, C is the catalytic subunit, [cAMP] is total cAMP concentration. A valid root was obtained as total catalytic subunit concentration of CAPK using Eq. A8, and is taken to be same in both liver and muscle.

In the current work, the following cascade-connecting complexes were neglected in the total interconvertable balance: complexes between CAPK catalytic subunits and inhibitor-1, phosphorylase kinase and glycogen synthase in the CAPK balance; inhibitor-1 complex with PP1 in the inhibitor-1 balance; PP1 complexes with phosphorylase kinase, phosphorylase and synthase in the PP1 balance; phosphorylase kinase complexes with phosphorylase and synthase in the phosphorylase kinase balance; liver glycogen phosphorylase complex with PP1 in the phosphorylase balance. We have verified the extent of formation of these complexes and they were found to be negligible compared to the corresponding total interconvertable enzymes. This assumption is valid when the dose-response curve of each target enzyme exceeds 90% phosphorylation [23].

### (II) Operating equations for covalent modification cycles [14] involved in regulation of glycogen synthesis and breakdown in the muscle

#### Cubic equation for phosphorylation-dephosphorylation cycle of Inhibitor-1



where *f_1 _*= *I*/*I*_*t*_, *I_t _*and *I *are total and unphosphorylated inhibitor concentration, *(PP2)_t _*is total phosphatase 2A, k_1 _and k_2 _are rate constants for phosphorylation and dephosphorylation of inhibitor-1 respectively. K_m1 _and K_m2 _are Michaelis-Menten constants for phosphorylation and dephosphorylation of inhibitor-1 respectively. From the constraint 0 <*f_1_*< 1, a valid root was obtained as a fractional unmodified inhibitor using Eq. A9. The fractional phosphorylated inhibitor (i.e. *I_p_*/*I_t_*) can then be obtained using the following relationship:



where *f_2 _*= *I_p_*/*I_t_*

#### Quadratic equation for allosteric interaction of phosphorylated phosphorylase with PP1 [34]



where *P_p _*is phosphorylated phosphorylase, *PP1.P_p _*is inactive PP1 and *Kd *is the dissociation constant.



where *(PP1)_t _*is total PP1 and *f_3 _*is fractional inactivated PP1 (i.e., (*PP1.I_p_*)/(*PP1*)_*t*_). The fractional free (active) species of PP1 (i.e. *f_4 _*= (*PP1*)/(*PP1*)_*t*_) can be estimated by *f_4 _*= *1-f_3_*.

#### Cubic equation for phosphorylation-dephosphorylation cycle of phosphorylase kinase



where *f_5 _*= *K*/*K_t_*, *K*_*t *_and *K *are total and unphosphorylated phosphorylase kinase concentration, k_3 _and k_4 _are rate constants for phosphorylation and dephosphorylation of phosphorylase kinase respectively. K_m3 _and K_m4 _are Michaelis-Menten constants for phosphorylation and dephosphorylation of phosphorylase kinase respectively. From the constraint 0 <*f_5 _*< 1, a valid root was obtained as a fractional unmodified phosphorylase kinase using Eq. A13. The fractional phosphorylated phosphorylase kinase (i.e. *K_p_*/*K_t_*) can then be obtained using the following relationship:



where *f_6 _*= *K_p_*/*K_t_*

#### Cubic equation for phosphorylation-dephosphorylation cycle of phosphorylase



where *f_7 _*= *P*/*P*_*t*_, *P*_*t *_and *P *are total and unphosphorylated phosphorylase concentrations, k_5 _and k_6 _are rate constants for phosphorylation and dephosphorylation of phosphorylase respectively. K_m5 _and K_m6 _are Michaelis-Menten constants for phosphorylation and dephosphorylation of phosphorylase respectively. From the constraint 0 <*f_7 _*< 1, a valid root was obtained as a fractional unmodified phosphorylase using Eq. A15. The fractional phosphorylated phosphorylase (i.e. *P*_*p*_/*P_t_*) can then be obtained using the following relationship:



where *f_8 _*= *P_p_*/*P_t_*

#### Cubic equation for phosphorylation-dephosphorylation cycle of glycogen synthase



where *f_9 _*= *S*/*S_t_*, *S*_*t *_and *S *are total and unphosphorylated glycogen synthase concentrations, k_7 _and k_8 _are rate constants for phosphorylation and dephosphorylation of glycogen synthase respectively. K_m7 _and K_m8 _are Michaelis-Menten constants for phosphorylation and dephosphorylation of glycogen synthase respectively. From the constraint 0 <*f_9 _*< 1, a valid root was obtained as a fractional unmodified glycogen synthase using Eq. A17. The fractional phosphorylated glycogen synthase (i.e. *S_p_*/*S_t_*) can then be obtained using the following relationship:



where *f_10 _*= *S_p_*/*S_t_*

A plot of fractional activation of catalytic subunit, inhibitor-1 (*f_2_*), phosphorylase kinase (*f_6_*), phosphorylase (*f_8_*) and glycogen synthase (*f_10_*) at different cAMP input concentrations in muscle is shown in Fig 2 of the main text.

### (III) Operating equations for covalent modification cycles involved in regulation of glycogen synthesis and breakdown in liver

In this case, glucose is considered to be the primary input to the enzyme cascades. Glucose-6-phosphate levels were estimated from various concentration of glucose using the following relationship:



where *g6pt *represents physiological (maximum) concentration of glucose-6-phosphate, *g6p *is the concentration of glucose-6-phosphate in relation to the concentration of glucose and *Kg *is an activation constant. Glucose concentration regulates intracellular cAMP levels through hormonal signals such as glucagon. The inverse relationship between glucose and cAMP levels is incorporated by the following equation:



where *cAMPt *represents the physiological (maximum) concentration of cyclic AMP, *cAMP *is the concentration of cyclic AMP in relation to the concentration of glucose and *Ki *represents the inhibitor constant. The superscript 2 and the parameters including *Ki*, *kg *and *kg2 *are suitably chosen so that glucose-6-phosphate and cAMP are relatively in the physiological range. cAMP is further taken as an input to CAPK activation. The analytic expression for this interaction is the same as given in Eq. A8.

#### Cubic equation for phosphorylation-dephosphorylation cycle of phosphorylase kinase



where *f_11 _*= *K*/*K_t_*, *K*_*t *_and *K *are total and unphosphorylated phosphorylase kinase concentrations, k_3 _and k_4 _are rate constants for phosphorylation and dephosphorylation of phosphorylase kinase respectively. K_m3 _and K_m4 _are Michaelis-Menten constants for phosphorylation and dephosphorylation of phosphorylase kinase respectively. From the constraint 0 <*f_11 _*< 1, a valid root was obtained as a fractional unmodified phosphorylase kinase using Eq. A21. The fractional phosphorylated phosphorylase kinase (i.e. *K*_*p*_*/K*_*t*_) can then be obtained using the following relationship:



where *f_12 _*= *K_p_*/*K_t_*

#### Equations for glucose and glucose-6-phosphate influence on enzyme cascades in liver

##### Glucose-6-phosphate inhibition of phosphorylase b phosphorylation



where K_m5 _is the Michaelis-Menten constant for phosphorylation of phosphorylase-b, K_m51 _represents K_m5 _modified by glucose-6-phosphate effects.

##### Activation of dephosphorylation of phosphorylase a by glucose



where K_m6 _is the Michaelis-Menten constant for dephosphorylation of phosphorylase a, K_m61 _represents K_m6 _modified by glucose effects. S2 is a multiplicative factor and kgi represents the activation constant.

#### Cubic equation for phosphorylation-dephosphorylation cycle of phosphorylase



where *f_13 _*= *P*/*P_t_*, *P*_*t *_and *P *are total and unphosphorylated phosphorylase concentrations, k_5 _and k_6 _are rate constants for phosphorylation and dephosphorylation of phosphorylase respectively. K_m5 _and K_m6 _are Michaelis-Menten constants for phosphorylation and dephosphorylation of phosphorylase respectively. From the constraint 0 <*f_13 _*< 1, a valid root was obtained as a fractional unmodified phosphorylase using Eq. A25. The fractional phosphorylated phosphorylase (i.e. *P*_*p*_*/P*_*t*_) can then be obtained using the following relationship:



where *f_14 _*= *P_p_*/*P_t_*

#### Quadratic equation for allosteric interaction of phosphorylated phosphorylase with PP1 [34]



where P_*p *_is phosphorylated phosphorylase, *PP1.I*_*p *_is inactive PP1 and *Kd *is the dissociation constant



where *(PP1)*_*t *_is total PP1 and *f_15 _*is fractional inactivated PP1 (i.e. (*PP1.P*_*p*_*)*/(*PP1)*_*t*_). The fractional free (active) species PP1 (i.e. *f_16 _*= (*PP1)*/(*PP1)*_*t*_) can be estimated by *f_16 _*= *1-f_15_*.

#### Equations for glucose and glucose-6-phosphate influence on enzyme cascades in liver

##### Activation of glycogen synthase dephosphorylation by glucose-6-phosphate



where K_m8 _is the Michaelis-Menten constant for dephosphorylation of synthase, K_m81 _represents K_m8 _modified by glucose-6-phosphate effects. S1 is a multiplicative factor and kg2 represents the activation constant.

##### Inhibition of glycogen synthase phosphorylation by glucose-6-phosphatase



where K_m7 _is the Michaelis-Menten constant for phosphorylation of synthase, K_m71 _represents K_m7 _modified by glucose-6-phosphate effects.

#### Cubic equation for phosphorylation-dephosphorylation cycle of glycogen synthase



where *f_17 _*= *S*/*S_t_*, *S*_*t *_and *S *are total and unphosphorylated glycogen synthase concentrations, k_7 _and k_8 _are rate constants for phosphorylation and dephosphorylation of glycogen synthase respectively. K_m7 _and K_m8 _are Michaelis-Menten constants for phosphorylation and dephosphorylation of glycogen synthase respectively. From the constraint 0 <*f_17 _*< 1, a valid root was obtained as a fractional unmodified glycogen synthase using Eq. A31. The fractional phosphorylated glycogen synthase (i.e. *S*_*p*_/*S*_*t*_) can then be obtained using the following relationship:



where *f_18 _*= *S_p_*/*S_t_*

A plot of fractional activation of catalytic subunit, phosphorylase kinase (*f_12_*), phosphorylase (*f_14_*) and glycogen synthase (*f_18_*) at different glucose, glucose-6-phosphate and cAMP concentrations in liver is shown in Fig 3 and 4 of the main text.

#### Parameters from the literature used for the simulations

##### Rate Constants

k1 = 1.4 sec^-1 ^rate constant for phosphorylation of inhibitor [48]

k2 = 0.01 sec^-1 ^rate constant for dephosphorylation of inhibitor [assumed]

k3 = 20 sec^-1 ^rate constant for phosphorylation of phosphorylase kinase [assumed]

k4 = 5 sec^-1 ^rate constant for dephosphorylation of phosphorylase kinase [assumed]

k5 = 20 sec^-1 ^rate constant for phosphorylation of Phosphorylase [42]

k6 = 5 sec^-1 ^rate constant for dephosphorylation of Phosphorylase [49]

k7 = 20 sec^-1 ^rate constant for phosphorylation of glycogen synthase [assumed]

k8 = 0.05 sec^-1 ^rate constant for dephosphorylation of glycogen synthase [assumed]

##### Michaelis-Menten constants

Km1 = 5 *μ*M for inhibitor phosphorylation [48]

Km2 = 0.7 *μ*M for dephosphorylation of Inhibitor [52]

Km3 = 0.4 *μ*M for Phosphorylation of phosphorylase kinase [assumed]

Km4 = 1.1 *μ*M for dephosphorylation of phosphorylase kinase [52]

Km5 = 10 *μ*M for phosphorylation of phosphorylase [25]

Km6 = 5 *μ*M for dephosphorylation of phosphorylase [47]

Km7 = 15 *μ*M for phosphorylation of glycogen synthase [assumed]

Km8 = 0.12 *μ*M for dephosphorylation of glycogen synthase [50]

Kd = 0.002 dissociation of PP1 and phosphorylated PP1 Inhibitor, and also phosphorylase a with synthase PP1 [47]

##### Total Concentrations

capkt = 0.25 *μ*M total R2C2 ie. cAMP dependent protein kinase, CAPK [3]

It = 1.8 *μ*M total Inhibitor concentration [3]

kt = 2.5 *μ*M total Phosphorylase kinase [35]

pt = 70 *μ*M total Glycogen Phosphorylase [3]

st = 3 *μ*M total Glycogen synthase [3]

PP1 = 0.25 *μ*M PTPase 1 [33]

PP2A = 0.025 *μ*M PTPase 2 [3]

##### Other parameters: (chosen as per various qualitative observations are in physiological ranges as given in [3,9,12,17,25,27,29,33,35,42,46-54])

k11 = 0.043 *μ*M Dissociation constant of cAMP [35]

k22 = 0.7 *μ*M Dissociation constant of cAMP [35]

ki = 100 *μ*M cAMP inhibition constant

campt = 10 *μ*M maximum cAMP [3]

kg = 349500 *μ*M activation constant of glucose-6-phosphate for synthase PP1

g6pt = 700 *μ*M maximum glucose-6-Phosphate [33]

kgi = 10000 *μ*M activation constant of glucose for phosphorylase phosphatase

s1 = 100 a multiplicative factor for glucose-6-phosphate effect on glycogen synthase dephosphorylation

kg2 = 500 *μ*M inhibition due to glucose-6-phosphate = 0.05 mM

s2 = 0.0010 a multiplicative factor for glucose effect on phosphorylase phosphatase

### Sensitivity analysis

The above parameter set was used for simulating the dose-response curves of the glycogen cascade system. To assess the sensitivity to variation in individual parameters, each parameter was varied over a 10-fold change while holding all other parameters constant. The response sensitivity is quantified using a Hill coefficient and is given in Table 1. The results indicate that at different parameter sets, the output responses of GP and GS are switch-like and display different degrees of signal amplification.
